# School Anxiety in Children and Adolescents with Chronic Pain

**DOI:** 10.1155/2017/8328174

**Published:** 2017-09-26

**Authors:** K. E. Jastrowski Mano

**Affiliations:** Department of Psychology, University of Cincinnati, Cincinnati, OH, USA

## Abstract

Anxiety is highly prevalent in pediatric chronic pain. This comorbidity has been explained by the presence of shared mechanisms underlying the development and maintenance of chronic pain and anxiety. Accumulating evidence demonstrates that school is a significant source of anxiety among youth with chronic pain and that anxiety contributes to school-related functional impairment in this population. This article reviews the cooccurrence of pediatric chronic pain and anxiety, identifies unique sources of heightened school anxiety among youth with chronic pain, and describes current approaches for assessing anxiety in pediatric pain settings. Highlighted by this review is the absence of a comprehensive evidence-based approach for assessing school anxiety in pediatric chronic pain. Given the psychometric limitations inherent to gathering data from a single source, recommendations for advancing measurement methods are provided. Novel approaches may be needed to shed more light on the way in which school anxiety is experienced in pediatric chronic pain.

## 1. Introduction

Emerging evidence has shown that anxiety is an important factor in pediatric chronic pain [1]. Anxiety is significantly more common in youth with chronic pain than in the general population [2–4]. This comorbidity is a critically important health problem. It is associated with poorer response to pain-focused cognitive-behavioral interventions [5] and increased risk for both chronic pain [6] and anxiety disorders in adulthood [7].

School can be a significant source of anxiety among pediatric chronic pain patients. School anxiety comprises several domains of academic and interpersonal distress, such as fears pertaining to academic performance, negative teacher evaluations, and peer relationships [8]. School anxiety is a significant concern among health care professionals as it is often linked with school avoidance behavior [9–12]. Though related, school anxiety and school avoidance (a term often used interchangeably with school refusal [13]) are distinct constructs. (Of note, though the terms school refusal and school avoidance have been used interchangeably, the term school* avoidance* has predominantly been used in the pediatric chronic pain literature [12]. School* refusal* is the more common term in the child anxiety literature [11] and has been applied more broadly to include youth exhibiting a variety of internalizing and externalizing (e.g., aggression, truancy) behaviors [13]. School avoidance, on the other hand, is primarily linked to internalizing (e.g., anxiety) rather than externalizing problems in the school environment [12].) Although anxiety is characterized by disruptions in multiple domains (e.g., cognitive, affective, and behavioral), the term* school anxiety* is typically used to describe the cognitive-affective (e.g., fear, worry) domain, whereas* school avoidance* is viewed as a behavioral manifestation of anxiety [11, 12] or a serious behavioral complication accompanying anxiety disorders in youth [14]. In other words, school avoidance is a pattern in which a child experiences severe anxiety related to school and thus avoids it, fostering frequent absenteeism as well as heightened anxiety [15]. School anxiety is not a recognized diagnostic category in the Diagnostic and Statistical Manual of Mental Disorders, Fifth Edition (DSM-5), at this time [16]. However, evidence of significant anxiety in the context of school warrants further diagnostic clarification, as it may be associated with a number of different DSM-5 clinical disorders (e.g., separation anxiety disorder, social anxiety disorder, or generalized anxiety disorder) [14, 17].

Despite overwhelming evidence of school anxiety among pediatric pain patients [18–20], research focused specifically on the measurement of school anxiety symptoms remains largely undeveloped. There is a need for more tailored assessment strategies targeting school anxiety in pediatric chronic pain [20–23] as clarifying the nature and extent of school anxiety in pediatric chronic pain has important implications for theory, assessment, and intervention.

This paper reviews the (i) comorbidity between pediatric chronic pain and anxiety, (ii) sources of heightened school anxiety, in particular, among youth with chronic pain, and (iii) existing approaches for assessing school anxiety. To guide future research on this understudied topic, suggestions for advancing the measurement of school anxiety are provided. This discussion includes a consideration of both traditional self-report methods as well as the potential role of novel, implicit assessment strategies toward improving our understanding of school anxiety.

## 2. Comorbidity between Chronic Pain and Anxiety

Anxiety symptoms are prevalent in pediatric chronic pain [3, 20] with upwards of 80% of chronic pain patients meeting criteria for an anxiety disorder based on structured diagnostic interviews [24, 25]. This has led to the call for routine screening for anxiety disorders in youth with functional abdominal pain [26] and in other pediatric pain populations [20].

Given that pain and anxiety are both associated with physiological arousal (e.g., accelerated heart rate, increased respiration rate, and muscular tension), it may be assumed that the high prevalence of anxiety symptoms in pediatric chronic pain is solely attributable to overlapping somatic symptoms (e.g., dizziness, difficulty breathing). However, this is not the case. Youth with chronic pain experience a wide range of anxiety disorder symptoms, including separation anxiety and social anxiety, which have fewer physiological symptoms included within the diagnostic criteria [20, 22]. For example, Tran and colleagues [20] found that 46% of pediatric chronic pain patients reported clinical elevations on at least one anxiety subscale on the Screen for Child Anxiety-Related Emotional Disorders (SCARED). Notably, the most common clinical elevations were on the school phobia, separation anxiety, and social anxiety subscales.

### 2.1. Shared Mechanisms of Pediatric Chronic Pain and Anxiety

The shared vulnerability model [27] posits that there is a similar underlying diathesis for chronic pain and anxiety. Specifically, the shared vulnerability model applies a diathesis-stress framework, postulating that individuals who are at increased risk for the development of chronic pain and/or an anxiety disorder share predisposing vulnerabilities, including anxiety sensitivity and reduced threshold for alarm, which give rise to particular negative emotional responses—namely, fear and anxiety—in the face of stressful events. Consequences of fear and anxiety, including attentional biases, avoidance behavior, and autonomic nervous system arousal, further contribute to the development of chronic pain, anxiety disorders, and their cooccurrence.

Though originally developed to explain the cooccurrence of posttraumatic stress disorder (PTSD) and chronic pain in adults, aspects of the shared vulnerability model have been well supported by research in pediatric populations. For example, anxiety sensitivity (i.e., fear of fear) has been shown to increase the risk for pain-related avoidance and disability in youth with chronic pain [28, 29]. In research with healthy children, anxious symptomatology is directly related to pain sensitivity; and anxiety sensitivity impacts pain intensity indirectly via its effects on pain-related anticipatory anxiety (e.g., [30]). Children with recurrent abdominal pain and children with anxiety disorders show indistinguishable laboratory stress responsivity [31, 32]. Notably, children with recurrent abdominal pain may be less likely to endorse trait anxiety and anxiety symptoms on* self-report* measures, despite demonstrating similar levels of state anxiety and physiological arousal in response to laboratory stressors [31].

Also included in the shared vulnerability model is avoidance behavior, as a characteristic of both anxious populations and chronic pain patients. The fear avoidance model is often used to explain avoidance in the context of pediatric chronic pain [33]. For instance, Simons and Kaczynski [33] describe similarities between youth with anxiety and pediatric patients with chronic pain, such that both groups tend to struggle with social (e.g., socializing with friends) and academic (e.g., attending school) developmental tasks. This often results in activity avoidance and, for many, significant disruptions in social and academic domains of functioning [3, 18, 34].

Finally, longitudinal research suggests that pain in childhood predicts anxiety in adulthood, even when pain symptoms have abated [7]. This is consistent with the shared vulnerability model such that common predisposing factors (e.g., anxiety sensitivity) and maintenance factors (e.g., avoidance behavior [35], attentional biases [36]) that are central to both conditions may connote heightened risk for the development and continuation of pain and/or anxiety across the lifespan.

## 3. School-Related Anxiety and Impairment in Pediatric Chronic Pain

Chronic pain in children and adolescents is associated with impaired school functioning in multiple domains [2, 3, 34, 35, 37–42]. In fact, school absence rates in pediatric chronic pain possibly exceed rates of absenteeism due to other chronic health conditions [34]. Youth with chronic pain who frequently experience periods of missed school often fall behind academically as a result of their pain symptoms [18, 23, 35, 37, 38]. Chronic pain patients also report lower grades and other maladaptive school-related behaviors following the onset of pain [37, 39].

Anxiety appears to be a key driving force behind the school disability and avoidant behavior that often characterizes youth with chronic pain [33, 40]. About one-third of youth with chronic pain exhibit anxiety-related school avoidance [23]. Anxiety is also a robust predictor of difficulties with keeping up academically and concentrating at school [12, 23, 35]. Anxiety has been shown to be a stronger predictor of functional disability than pain severity [23]. Further, in the context of high levels of anxiety and worry, pain is unrelated to school functioning [40] or overall functional disability [33]. Thus, research suggests that anxiety plays a central role in maintaining school disability by driving school avoidant behavior, thus perpetuating a cycle of avoidance that, in turn, further heightens anxiety [39]. It is not surprising that the coupling of anxiety and pediatric chronic pain creates an especially high risk for school-related disability [22].

Several studies have demonstrated seasonal patterns of pain and anxiety complaints, indicating that these difficulties frequently cooccur, may influence each other, and fluctuate corresponding to the school year [43–46]. For example, Saps and colleagues [46] found that consultations for anxiety and complaints about abdominal pain were most common in the winter months, declining throughout the summer months and steadily rising again in the fall. They speculated that the winter dominance of pain-related and psychiatric complaints in children is attributable to school-related anxiety and stress. Showing a very similar pattern—and drawing the same conclusion—a recent study found that the volume of parent phone calls made to a pediatric pain clinic pertaining to headache and abdominal pain was approximately three times higher during the winter [47].

## 4. Specific Sources of School Anxiety among Chronic Pain Patients

School is a source of anxiety among youth with chronic pain, supported by the aforementioned evidence of high rates of anxiety and school avoidance exhibited in this population. School anxiety plays an important role in both getting to school and functioning while at school [23, 35]. For example, school anxiety frequently peaks on school day mornings—manifesting in a variety of anxious and somatic symptoms—resulting in parents deciding to let their child stay home from school [12]. When at school, youth with chronic pain may worry about whether pain symptoms will negatively influence their test-taking performance or bother them while trying to participate in other academic or social activities.

Though chronic pain patients understandably have a heightened sensitivity to experiencing physical symptoms when at school (e.g., what if my stomach starts to hurt after I eat my lunch?), school represents a myriad of other sources of anxiety as well. As is to be expected during childhood and adolescence, some pediatric chronic pain patients likely experience some degree of anxiety regarding their grades, performance on standardized tests, and other aspects of their academic performance. Not surprisingly, youth with chronic pain often report feeling as though pain has negatively impacted their school success [39]. It makes sense then that youth with chronic pain, who not only need to grapple with the typical test-related performance stress, also have the additional burden of worrying about the degree to which pain symptoms may distract them during important academic situations. To date, there is no published data establishing the degree to which youth with chronic pain struggle with specific types of academic anxiety, such as test anxiety or performance-related anxiety (e.g., giving speeches in front of class).

The social aspects of school likely also give rise to anxiety. Research suggests that approximately 20 to 25% of youth with chronic pain report elevated social anxiety scores [20]. Likewise, given the high prevalence of public speaking anxiety in childhood [48] it is probable that a good number of chronic pain patients experience heightened fear of speaking in front of others at school—which may occur in the context of class presentations, but also in subtler forms such as raising one's hand during class to ask the teacher a question. Finally, youth with chronic pain may also worry about teachers' perceptions, perhaps sensing that teachers are unsupportive or misunderstand the pain problem. Such concerns are not unwarranted, as teachers have been shown to lack a biopsychosocial framework for understanding chronic pain in childhood [49]. This is concerning given how common it is for chronic pain patients to need to initiate conversations with one or more teachers in regard to classroom accommodations (e.g., requesting permission to leave class to go to the nurse's office), assignment accommodations (e.g., requesting deadline extensions), or school absences.

Social-evaluative concerns are developmentally appropriate during childhood and adolescence. However, chronic pain patients may face additional challenges, such as feeling different than peers due to having a pain condition or finding it challenging to explain their pain condition or reasons for school absences to peers. Children and adolescents with chronic pain may have fewer friends, be more socially isolated, and experience higher rates of peer victimization compared to youth without pain [50]. To the degree that youth with chronic pain perceive social situations at school as threatening, over time, they may develop fear-related avoidance behavior toward school [33].

Forgeron et al. [51] found social information processing differences between youth with chronic pain and healthy controls. Specifically, adolescents with chronic pain showed heightened sensitivity to vignettes depicting potentially nonsupportive social situations. When asked to envision themselves as a healthy friend in vignettes, adolescents with chronic pain indicated that they would have enacted more supportive behaviors toward a chronic pain vignette character. The authors suggested that youth with chronic pain may expect more supportive behaviors from their friends and when they perceive friends at school as being unsupportive may distance themselves socially and avoid particular social situations [51].

In a recent study examining adolescents' interpretation biases, it was found that those who reported greater pain catastrophizing and more recent pain complaints endorsed more negative interpretations (and rejected more benign interpretations) of ambiguous situations regarding pain and bodily threat [52]. Interestingly, these adolescents showed the same pattern for ambiguous* social situations*, suggesting a generalized rather than pain-specific interpretation pattern—and a pattern that would be expected of those experiencing anxiety in general or social anxiety specifically. Though speculative, it may be that youth who are vulnerable to interpreting ambiguous situations as threatening tend to apply such interpretations broadly. This would be consistent with the shared vulnerability model of chronic pain and anxiety [27, 53], as well as research suggesting that youth with chronic pain have more difficulty attending to and interpreting social cues [54]. In other words, youth at increased risk for developing negative bodily threat interpretations—a risk factor for the development of chronic pain—may exhibit similar cognitive biases that increase their risk for the development and/or maintenance of anxiety disorder symptoms.

Parent influences are also important to consider. Robust evidence demonstrates that parental protectiveness in response to pain confers risk for school impairment in youth with chronic pain [55] and has been shown to mediate the relationship between parental pain catastrophizing and child school attendance rates and general school impairment [56]. Furthermore, there is evidence to suggest that parental distress operates, at least in part, through amplification of child anxiety. For example, etiological studies suggest that parental anxiety is a crucial factor in the development of childhood anxiety [57, 58] and has been shown to predict children's physiological reactivity following stress [59]. These findings underscore the importance of evaluating parental anxiety and parental responses to children's school anxiety symptoms, as both are likely important contributors to the child's affective and behavioral responses to stressors occurring in the school environment.

In summary, school represents a context in which youth with chronic pain experience significant anxiety and school-related functional disability. There are many unique sources of school-related anxiety, such as fear of academic failure or inability to keep up with demands, fear of negative peer evaluation, and fear of experiencing physical symptoms at school. Knowing that youth with chronic pain generally experience elevated school anxiety is not specific enough to guide intervention efforts. Thus, all potential sources of school anxiety should be assessed because each one may be a unique treatment target [22, 23].

## 5. Assessment of School Anxiety: Current Practice and Limitations

The primary measurement issue stalling efforts to understand school anxiety in pediatric pain is simple—there are no instruments designed for this particular purpose. A measure specifically focused on school anxiety has yet to be developed or normed for use in pediatric pain settings. Thus, despite growing recognition of the importance of evaluating school anxiety in pediatric chronic pain [20, 22], clinicians and researchers lack a comprehensive evidence-based approach for doing so.

Current methods for assessing anxiety among pediatric pain patients consist of using either broadband measures of psychopathology symptoms that include one or more anxiety-related subscales, or narrow-band anxiety measures, such as the Multidimensional Anxiety Scale for Children (MASC) [60], Revised Children's Manifest Anxiety Scale (RCMAS) [61], or the Screen for Child Anxiety-Related Emotional Disorders (SCARED) [62]. One key concern regarding this practice is that although these anxiety measures have demonstrated strong psychometric properties in the general population or in treatment-seeking psychiatric samples, few have been validated for use in pediatric settings [63]. This calls into question whether current measures of anxiety—regardless of their psychometric properties in other samples—are appropriate for youth with pediatric chronic pain.

Existing anxiety measures also lack adequate content validity for the assessment of* school* anxiety specifically. For example, the MASC contains only two items that explicitly mention school as a context for anxiety symptoms (“I worry about being called on in class” and “I try hard to obey my parents and teachers”). Other MASC items may apply to how the child feels at school but do not specifically refer to school situations or school peers (e.g., “I worry about what other people think of me”). Similarly, though the SCARED has shown good evidence of internal consistency and construct validity when used in a treatment-seeking pediatric chronic pain sample [22], the primary caveat for its usage is its lack of an appropriate school anxiety subscale. The SCARED School Phobia subscale showed poor internal consistency—likely due to problems with content validity, in terms of both item content and scope. Specifically, the School Phobia subscale comprises only four items; thus it lacks the necessary breadth to adequately measure a multifaceted construct like school anxiety. When used in pediatric pain settings, the fact that some School Phobia items also mention specific pain symptoms is especially problematic [22].

## 6. Future Directions in the Assessment of School Anxiety

Current assessment approaches limit our understanding of the precise fears of youth with chronic pain. This has led to a call for more research to address gaps in the assessment of school anxiety [20–22]. Given the limitations of using broad anxiety measures to gauge school anxiety, one possibility would be to expand the school-related content of existing self-report anxiety measures, such as the SCARED. An arguably better solution would be to develop a new, multifaceted school anxiety measure—one that includes items assessing fears pertaining to academic performance, such as test anxiety and falling behind on assignments, negative teacher evaluations, and peer relationships—that could be validated for use in pediatric chronic pain.

Because anxiety in pediatric chronic pain may manifest itself in ways that existing instruments were not developed to evaluate, it would be advantageous to gather input from pain patients to ensure that item content is relevant to and representative of the way in which school anxiety is experienced. For example, though not typically included in measures of school anxiety in nonmedical populations, it may be clinically relevant to include content pertaining to fears about pain symptoms interfering with academic performance or concerns about having to ask the teacher to go the nurse's office. This focus on content validity would help determine the most relevant content to include, the irrelevant content to exclude, and how to best achieve content balance (i.e., avoiding excessive over- or underemphasis of some aspects of social anxiety) [64]. These are important issues regardless of whether an existing anxiety self-report measure is modified or a completely new instrument is developed.

Existing measures of related constructs, such as school refusal, may also be relevant for capturing behavioral complications associated with school anxiety in pediatric chronic pain. The School Refusal Assessment Scale-Revised (SRAS-R) [65] measures four hypothesized functions of school refusal, including avoidance of stimuli that provoke negative affect and escape from aversive social situations. Similarly, the School Refusal section of the Anxiety Disorders Interview Schedule (ADIS-IV) [14] assesses whether a child has difficulty going to or staying at school and, if so, queries potential reasons for school refusal. Given that these measures have not been validated for use with pediatric pain patients, or more broadly, for youth with cooccurring anxiety and chronic medical conditions, items will likely need to be tailored. For instance, the SRAS-R may require the modification and/or addition of content that more clearly distinguishes school avoidance due to pain symptoms versus other (nonpain) factors. Some youth with chronic pain may report that school avoidance occurs exclusively in response to pain symptoms, whereas others may be able to identify academic and social factors that also keep them from going to school (e.g., they feel as though they do not have many friends at school; they are afraid of tests or riding the school bus).

It may also prove useful to explore other measures that have been developed particularly for pediatric chronic pain that may capture some aspects or correlates of school anxiety. Measures of pain anxiety, pain catastrophizing, and fear of pain, while not focused on school situations specifically, may be helpful when developing school anxiety measures in this population. Such measures would make it possible to distinguish youth whose fear and avoidance are limited to the school context from those who manifest a more generalized pattern of pain avoidance. This would strengthen the discriminant validity of school anxiety measurement.

Given the psychometric issues inherent to gathering data from a single source [66], it is imperative to include multiple perspectives. Teachers and other key school personnel are uniquely positioned to provide insights based on their direct observations of the child's behavior at school, including how the child functions in the classroom and in his or her interactions with peers. Parent-proxy reports are valuable in discerning the child's school-related anxiety symptoms at home, such as heightened anxiety on school day mornings compared to weekends, distress about upcoming tests, and difficulty keeping up with homework assignments.

Novel measurement approaches may also be needed due to the numerous challenges inherent to assessing school-related fears in pediatric chronic pain with self-report methodology. For instance, youth with chronic pain often underreport anxiety [21, 33, 38]. Logan and colleagues [21] found that 31% of their pain sample likely minimized anxiety on a self-report measure by responding in a socially desirable manner. As mentioned previously, children with chronic pain have shown to be less likely to* self-report* anxiety but responded to a threatening stressor with the same degree of anxiety and physiological stress reactivity as children with anxiety disorders [31]. Moreover, youth often verbally report wanting to attend school and experiencing minimal anxiety, despite exhibiting school avoidance behavior [35]. It has been argued that some youth with chronic pain who experience school anxiety may have difficulty identifying or articulating specific precipitating stresses. Other youth may be unaware of the extent of their school anxiety, identifying pain symptoms as the* sole* reason for not being able to attend school [12].

### 6.1. Potential Utility of Novel Implicit Measures

Multiple methods are needed to evaluate different facets of any problem. Given the limitations of self-report measures of school anxiety in the context of pediatric pain, novel* implicit* measures may represent an important assessment tool to circumvent youths' difficulty with overtly discussing school-related fears and reason(s) for school avoidance. Implicit measures, in conjunction with subjective self-reports, have the potential to shed new light on the way in which school anxiety is experienced in pediatric chronic pain.

Evaluation of implicit school-related* attentional biases* may be a useful approach in discerning the most salient facets of school anxiety. Attentional biases involve implicit, preferential tendencies to orient attention to particular threatening stimuli [67]. Various cognitive theories (e.g., attentional control theory) assert that anxiety is characterized by an attentional bias toward personally relevant, threatening stimuli [68]. Attentional biases have been implicated in both the development and maintenance of anxiety disorders [69–71]. In other words, attentional biases have been shown to be associated with current anxiety symptoms and also confer risk for the development of anxiety [72]. Though extant theories make different assumptions about the precise role that attentional biases play in anxiety (e.g., whether they play a causal role or not), available evidence suggests that attentional biases and anxiety are mutually maintaining [73].

Attentional biases represent a potential mechanism underlying the cooccurrence of chronic pain and anxiety [27, 53]. Attentional biases in the context of school, though implicit, may influence school functioning even when a particular behavior (e.g., school avoidance; [12]) stands in opposition to long-term goals (resuming or maintaining adaptive school functioning). To date, only* pain*-related attentional biases have been examined in pediatric chronic pain [54, 74, 75]. There remains a dearth of research considering other types of attentional biases that may be relevant to pediatric chronic pain patients. When considering* school* as a source of threat in the lives of youth who experience chronic pain, attentional biases for stimuli that become associated with pain and anxiety, such as school-related triggers, may become risk factors for the perpetuation of school anxiety* and* chronic pain symptoms over time [36, 76].

Studies characterizing the mechanism(s) underlying school-related attentional biases exhibited by youth with chronic pain may be important. Eye-tracking methods—which are able to continuously and directly measure attention—are able to assess various patterns of attention. For example, school anxious youth may demonstrate an attentional bias driven by an* initial orienting bias* that involves a constant visual search of the environment for school-related threat, such that the child's attention is more quickly captured by school-related threatening stimuli relative to other stimuli. Here, the child shows hypervigilance to school threat stimuli. An attentional bias may also be driven by an* attention maintenance bias* involving difficulty disengaging from school-related threat, wherein, after the child fixates upon a school-related stimulus, he/she is unable to shift attention away from the threatening stimulus. This pattern suggests excessive cognitive processing of threat. Finally, an attentional bias may be characterized by a* vigilance-avoidance* pattern that involves an orienting bias toward school-related threat (momentary fixation upon a stimulus) followed by avoidance of the stimulus. Here, the child scans the environment for a threat stimulus, and once they find it, they actively avoid it.

Notably, all three patterns have been identified in the literature. While the above hypothetical scenarios share many facets of cognitive processing, they are distinct enough to warrant scientific inquiry so as to advance our understanding of the specific pattern(s) of school-related attentional biases (e.g., hypervigilance or vigilance-avoidance) that may exist in pediatric chronic pain. It is possible that particular patterns of school-related attentional biases connote a higher risk for school anxiety. For example, based on recent work in test anxiety [77], it is plausible that youth with chronic pain who show a pattern of early attentional engagement followed by avoidance of school-related threat are at particular risk for school anxiety.

## 7. Conclusion

Both chronic pain and school anxiety in childhood are associated with concurrent and long-term impairment [41, 78–80]. An evidence-based approach for assessing school anxiety in pediatric chronic pain is needed, as existing measures fail to adequately capture this specific form of anxiety [20, 22, 81]. More research is needed to improve upon or develop new self-report measures of school anxiety. Implicit measures, in conjunction with subjective self-reports, may have the potential to shed new light on the way in which school anxiety is experienced in pediatric chronic pain.
